# Clinical application of whole transcriptome sequencing for the classification of patients with acute lymphoblastic leukemia

**DOI:** 10.1186/s12885-021-08635-5

**Published:** 2021-08-02

**Authors:** Wencke Walter, Rabia Shahswar, Anna Stengel, Manja Meggendorfer, Wolfgang Kern, Torsten Haferlach, Claudia Haferlach

**Affiliations:** 1 MLL Munich Leukemia Laboratory, Max-Lebsche-Platz 31, 81377 Munich, Germany; 2 Department of Hematology, Hemostasis, Oncology, and Stem Cell Transplantation, Hannover Medical School, 30625 Hannover, Germany

**Keywords:** Patient classification, Whole transcriptome sequencing, Fusion transcript calling, Gene expression profiling

## Abstract

**Background:**

Considering the clinical and genetic characteristics, acute lymphoblastic leukemia (ALL) is a rather heterogeneous hematological neoplasm for which current standard diagnostics require various analyses encompassing morphology, immunophenotyping, cytogenetics, and molecular analysis of gene fusions and mutations. Hence, it would be desirable to rely on a technique and an analytical workflow that allows the simultaneous analysis and identification of all the genetic alterations in a single approach. Moreover, based on the results with standard methods, a significant amount of patients have no established abnormalities and hence, cannot further be stratified.

**Methods:**

We performed WTS and WGS in 279 acute lymphoblastic leukemia (ALL) patients (B-cell: *n* = 211; T-cell: *n* = 68) to assess the accuracy of WTS, to detect relevant genetic markers, and to classify ALL patients.

**Results:**

DNA and RNA-based genotyping was used to ensure correct WTS-WGS pairing. Gene expression analysis reliably assigned samples to the B Cell Precursor (BCP)-ALL or the T-ALL group. Subclassification of BCP-ALL samples was done progressively, assessing first the presence of chromosomal rearrangements by the means of fusion detection. Compared to the standard methods, 97% of the recurrent risk-stratifying fusions could be identified by WTS, assigning 76 samples to their respective entities. Additionally, read-through fusions (indicative of *CDKN2A* and *RB1* gene deletions) were recurrently detected in the cohort along with 57 putative novel fusions, with yet untouched diagnostic potentials. Next, copy number variations were inferred from WTS data to identify relevant ploidy groups, classifying an additional of 31 samples. Lastly, gene expression profiling detected a *BCR*-*ABL1*-like signature in 27% of the remaining samples.

**Conclusion:**

As a single assay, WTS allowed a precise genetic classification for the majority of BCP-ALL patients, and is superior to conventional methods in the cases which lack entity defining genetic abnormalities.

**Supplementary Information:**

The online version contains supplementary material available at 10.1186/s12885-021-08635-5.

## Background

Transcriptome sequencing, usually gene expression arrays, has been a well-established diagnostic tool to characterize and quantify gene expression profiles and to detect fusion transcripts for many years. The development of RNA sequencing (RNA-Seq), including polyA-selected and whole transcript sequencing (WTS), has made it possible to broaden the analytical spectrum to study multiple transcriptional events (e.g., chimeric transcripts, isoform switching, expression, etc.) with a single approach. Compared to the expression arrays, RNA-Seq offers single base pair resolution, and considerably less background noise, providing hence, a relatively unbiased analysis of the transcriptome. Although guidelines have been established to allow for precise and context-dependent data analysis [1], no gold standard exists for any of the preprocessing steps or the downstream analyses. Hence, integrating RNA-Seq into the necessarily rigid quality standards of clinical diagnostic workflows is challenging. However, the multifaceted output of the assay can greatly benefit clinical diagnostics as indicated by various studies [2–4] and reviews [5, 6].

Acute lymphoblastic leukemia (ALL) is a heterogeneous hematological neoplasm when considering the clinical and genetic characteristics [7]. The World Health Organization (WHO) recognizes nine different sub-entities within the BCP-ALL with recurrent genetic abnormalities [8], including 4 groups characterized by the specific translocations, which result in the formation of aberrant chimeric transcripts detectable by RNA-Seq fusion calling (*BCR-ABL1*, *KMT2A*-rearranged, *ETV6-RUNX1*, *TCF3-PBX1*). Additional entities are characterized by abnormalities in chromosome numbers, or by partial amplifications: BCP-ALLs with hyperdiploidy, hypodiploidy or intrachromosomal amplification of chromosome 21 (iAMP21). More recently, further distinct ALL subtypes, including *BCR-ABL1*-like and *ETV6-RUNX1-*like ALL, were identified based on their gene expression profiles [9–11]. *BCR-ABL1*-like was included in the WHO classification of 2017, as a provisional entity based on treatment and prognostic implications that are associated with this high-risk subtype [8].

Currently, the diagnosis of ALL patients requires various analyses encompassing morphology, immunophenotyping, molecular analysis of gene fusions and mutations, and detection of numerical and structural abnormalities based on chromosomal banding analysis (CBA) and fluorescence in situ hybridization (FISH) [12]. With WTS parallel analysis of gene expression profiles, fusion transcripts and copy number changes becomes feasible, leading to an in-depth characterization of a patients’ genetic profile as a basis for disease classification based on the data set of a single approach.

Nevertheless, studies that comprehensively assessed all the transcriptional events in a clinical setting are scarce. In the current study, we performed detailed WTS analysis in 279 patients with newly diagnosed ALL of B- and T-lineage, to explore the complete diagnostic potential of WTS for the genetic characterization of ALL and its applicability in routine practice.

## Methods

### Patients and samples

Two hundred seventy nine patients with newly diagnosed ALL, sent to MLL Leukemia Laboratory between 03/2006–01/2017 for diagnostic work-up, were selected based on sample availability for WTS and WGS. ALL diagnosis was established based on morphology, immunophenotype, and cytogenetics, as previously published [13–15]. The cohort comprised 115 female (41%) and 164 male (59%) patients, with a median age of 49 years (range 0.1–93 years) at diagnosis (Additional file 3: Table S1). The patients showed B-cell precursor (BCP-ALL; *n* = 211) or T-cell precursor immunophenotype (T-ALL; *n* = 68). For WTS analysis 64 (45% female, 55% male), healthy individuals were sequenced as controls.

### CBA, FISH, array-CGH

CBA was performed for all 279 cases as previously described [13]. Classification of chromosomal aberrations and karyotypes was performed according to the ISCN 2016 guidelines [16]. The FISH probes used in diagnostic work-up were selected based on recommendations, aberrations detected in CBA, and the availability of probes. Array-CGH analyses were carried out for 123 cases (4x180K microarray slides, Agilent Technologies, Santa Clara, CA). The design was based on UCSC hg19 (NCBI Build 37, February 2009).

### Library preparation, sequencing, and data preprocessing

Library preparation was done as previously described [17]. In brief, genomic DNA and total RNA were extracted from lysed cell pellet of diagnostic bone marrow (*n* = 196) or peripheral blood (*n* = 83). Two hundred fifty ng of high-quality RNA were used as input for the TruSeq Total Stranded RNA kit (Illumina, San Diego, CA, USA). WGS libraries were prepared from 1 μg of DNA with the TruSeq PCR free library prep kit (Illumina). For WTS, 101 bp paired-end reads were produced on a NovaSeq 6000 system with a median yield of 68 million cluster per sample. WGS libraries were sequenced on a NovaSeq 6000 or HiSeqX instrument with 90x coverage and 150 bp paired-end sequences (Illumina). FASTQ generation was performed applying Illumina’s bcl2fastq software (v2.20). Using BaseSpace’s RNA-seq Alignment app (v2.0.1) with default parameters, reads were mapped with STAR aligner (v2.5.0a, Illumina) to the human reference genome hg19 (RefSeq annotation). Reads from WGS libraries were aligned to the human reference genome (GRCh37, Ensembl annotation) using the Isaac aligner (version 03.16.02.19).

### DNA and RNA-based genotyping

The haplotype caller was used to identify the variant allele frequencies of 50 single nucleotide polymorphisms (SNP) [18], following the best practice guidelines of GATK4 [19]. The allele concordance score, defined as the ratio between the number of identical alleles and the total number of alleles, was computed for all pairwise comparisons to identify the best WGS match to each WTS profile. WTS coverage of the chromosomal region of the various SNPs were assessed by samtools [20] depth command. Only the SNPs with at least 5 reads in a patients’ WTS data, were used for the comparisons.

### Structural variant and copy number variant detection on WGS data

For WGS, no sample specific normal tissue was available. A sequencing-platform and gender specific genomic DNA from a mixture of multiple anonymous donors (Promega, Fitchburg, WI, USA) was used as a normal in a tumor/unmatched normal workflow to call structural variants (SV; aberrations with > 50 bp in size) with Manta (v0.28.0). For somatic copy number variations (CNV), GATK4 was used following the Broad’s recommended best practices with a panel of normals. Specific gene deletions (*IKZF1, CDKN2A, RB1*) were identified by matching SV and CNV calls within the respective region.

### Gene expression analysis

Estimated read counts per gene were obtained from Cufflinks 2 (version 2.2.1). Non expressed genes were filtered out (< 2 counts). Raw counts were normalized by applying the Trimmed mean of M-values method from the edgeR package [21], producing log_2_ CPM values. t-SNE plots were generated with the R package Rtsne (https://github.com/jkrijthe/Rtsne). Circos plots were generated with RCircos [22]. Venn diagrams were produced with BioVenn [23]. The remaining plots were generated with ggplot2 (ref. [24]). For *BCR*-*ABL1*-like expression analysis the median expression profiles of selected genes from 40 *BCR*-*ABL1* positive and 65 *BCR*-*ABL1* negative cases were used as references. Classification was done based on the minimal Euclidean distance.

### Fusion calling on WTS data

Arriba (https://github.com/suhrig/arriba), STAR-Fusion [25], and MANTA [26] were selected for fusion calling. All the algorithms were used with default settings, except for STAR-Fusion --min_FFPM, which was set to zero, to include all candidate fusion transcripts independent of estimated expression. Fusions were only considered for further analysis, if they were called by at least two callers, could be confirmed by WGS, and were not detected in control samples. Putative novel fusions were queried against the Mitelman Database of Chromosome Aberrations and Gene Fusions (https://mitelmandatabase.isb-cgc.org/) and ChimerDB [27].

### Copy number inference on WTS data

The copy number states of the autosomes were inferred from raw gene counts with the ‘import-rna’ option of the software package CNVkit [28]. The obtained results were further filtered, and only the calls with a weight > 15 were considered. Individual calls were aggregated per chromosome. A copy number state was considered as aberrant, if the log_2_ value was > 0.15. Samples with > 3 copy number changes (chromosome gains or losses) were selected as potential low hypodiploidv/near triploidy and high hyperdiploidy cases. Samples with either loss of ≥5 chromosomes or the specific loss of chromosomes 3, 7, 13, and 17, were assigned to the low hypodiploid/near-triploid group. Cases were categorized as high hyperdiploid if at least 2 of the following chromosomes were gained: 4, 6, 10, 14, 17, 18, and 21.

### Selected small nucleotide variant analysis

The WTS data was evaluated for SNVs in *CRLF2*, *DUX4*, *JAK2*, *KRAS*, *NRAS*, *PAX5* and *TP53*. Variants were called with the Isaac Variant Caller (version 2.3.13) and only the passed variants with a matching call in WGS data were included. For the WGS data, a gender-matched reference DNA was used for unmatched normal variant calling with Strelka2 (version 2.4.7).

## Results

### SNP profiles verify correct WGS/WTS pairing

A recently established SNP panel for both DNA and RNA-based genotyping [18], was used to identify potential sample mix-ups and/or contaminations between the WGS and WTS samples. The allele concordance score (ranging from 0 to 1; Patients & Methods) was used to identify the best matching WGS sample for each WTS profile. For 278/279 cases, the best matching WGS sample belonged to the same patient as the WTS sample with a minimal allele concordance score of 0.81 (Additional file 4: Table S2). However, for one of the samples, a substantial number of SNPs showed divergent VAFs between the WTS and WGS datasets, resulting in the elimination of the patients’ dataset.

### Gene expression reliably segregates BCP-ALL from T-ALL patients

**Fig. 1:**
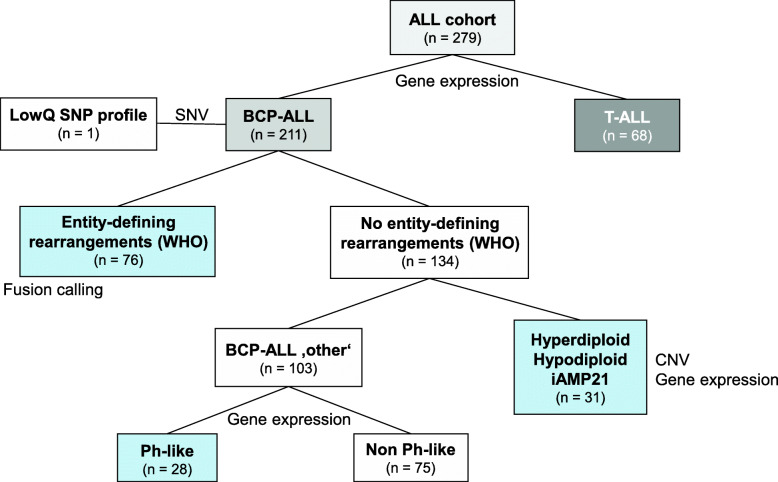
Classification tree. Design of the stepwise classification approach and distribution of the patients. First, DNA and RNA-based genotyping (SNV) was used to ensure correct WTS-WGS pairing, excluding one sample with low concordance. Gene expression analysis was used to distinguish between BCP-ALL or the T-ALL group. Subclassification of BCP-ALL samples was done progressively, assessing first the presence of entity-defining rearrangements (WHO) by the means of fusion detection. Next, copy number variations were inferred from WTS data to identify relevant ploidy groups. Lastly, gene expression profiling was used to identify BCR-ABL1-like signatures. ALL: Acute lymphoblastic leukemia; BCP-ALL: B-cell precursor acute lymphoblastic leukemia; CNV: copy number variation; iAMP21: intrachromosomal amplification of chromosome 21; LowQ: low quality; Ph: *BCR*-*ABL1*; SNP: single nucleotide polymorphism; T-ALL: T-cell acute lymphoblastic leukemia; WHO: world health organization

The samples were classified by WTS data following the classification tree depicted in Fig. 1. The initial classification step comprised the assignment of the samples to either the T, or the B lineage. As expected, the gene expression data could be used to reliably differentiate between BCP-ALL and T-ALL samples, based on the expression levels of 14 described markers (Additional file 1: Fig. S1A, Additional file 5: Table S3) [29]. Both lineages comprise different subtypes, characterized by the expression of various differentiation markers, and thereby defining the maturation state. Mapping the sample subtype classification (immunophenotyping data, Additional file 3: Table S1) to the two groups showed that the clusters within the groups fitted loosely to these subtypes (Additional file 1: Fig. S1B). However, further subclassification based on gene expression data, is rather challenging, and only for *CD10* (common B-ALL) and *CD1A* (thymic T-ALL) discriminative power of the expression data could be detected (Additional file 1: Fig. S1C). The *CD10* and *CD1A* expression values obtained from WTS correlated well with the percentage of positive cells obtained from immunophenotyping (R^2^ = 0.75; Additional file 1: Fig. S1D).

### Fusion calling identifies subgroup defining rearrangements with high accuracy

Following the segregation of the samples into the two lineages, the BCP-ALL samples are further subclassified by the identification of recurrent risk-stratifying gene fusions. The median of fusions per patient was 1 (range 0–8). In total, 100 unique fusion transcripts were called in the BCP-ALL cohort. Fourteen fusion transcripts occurred recurrently, while 86 fusion transcripts could only be detected in a single patient. 56% of these fusion transcripts involved genes located on the same chromosome (48/86; intra-chromosomal fusion) and 44% were caused by structural rearrangements between two different chromosomes (38/86; inter-chromosomal fusion). Based on the results from CBA, 41 *BCR*-*ABL1*, 23 *KMT2A*-*AFF1*, 5 *ETV6*-*RUNX1*, and 4 *TCF3*-*PBX1* fusions were detected in the cohort. The fusion calling based on WTS data identified 97% of these fusions (Table 1) with no false positives. In addition, WTS detected three other known fusion partners of *KMT2A (MLLT10*, *MLLT1*, and *USP2),* each in a different case, but missed one *KMT2A*-*EPS15* fusion, assigning 76/211 BCP-ALL samples to their respective subgroups. For 15% of these samples an additional fusion transcript was called. Except for *WDR37*-*TBRG1*, which co-occurred with the reciprocal of the *KMT2A*-*MLLT10* fusion transcript and involved the same chromosomes, but with breakpoints further apart, all of the additional fusion transcripts were intra-chromosomal (Additional file 6: Table S4).

**Table 1:** Comparison of detected fusion transcripts by RNA-Seq to expected translocations by chromosome banding analysis (CBA)

	CBA	RNA-Seq	RNA-Seq/CBA [%]
***BCR-ABL1***	41	40	98
***ETV6-RUNX1***	5	4	80
***KMT2A-AFF1***	23	23	100
***TCF3-PBX1***	4	4	100

### Broadening the spectra of fusion transcripts

**Fig. 2:**
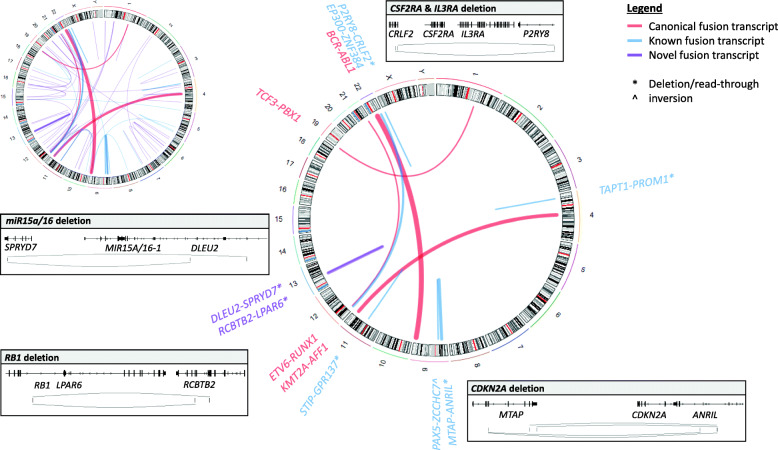
Fusion landscape of BCP-ALL. The Circos plots depict the spectra of identified fusion transcripts. Top left: all fusion transcripts; right: recurrent fusion transcripts. The line width of the central links correlates with the frequency of the fusion transcript. The colors represent the status of the fusion transcripts: Red - canonical, blue - known, purple - novel. * indicate deletion/read-through events, ^ inversions. Rectangles show fusion breakpoints of selected deletion/read-through events accompanied with gene annotations

In addition to the subtype defining rearrangements, among the BCP-ALLs we identified well characterized fusions involving *ZNF384* (*n* = 8), *PAX5* (*n* = 3), and two fusion transcripts containing *NUTM1*: *BRD9*-*NUTM1*, which has been described in infant ALLs [30], and the novel fusion *CHD4*-*NUMT1.* We also identified one case with an *EBF1*-*PDGFRB* fusion, which arose from an interstitial 5q33 deletion (WGS data), and another, with a *TCF3*-*HLF* fusion transcript. Known fusion transcripts in the T-ALL cohort mainly involved *MLLT10* (n = 3) and genes encoding for proteins of the nuclear pore complex (*n* = 5). Interestingly, we also identified recurrent read-through events, such as *MTAP-ANRIL* (*n* = 15), *RCBTB2*-*LPAR6* (*n* = 12), *P2RY8*-*CRLF2* (n = 3) and *DLEU2*-*SPRYD7* (n = 3) in both groups. Even if the fusions themselves are most likely not biologically active, *MTAP-ANRIL* has been detected in melanoma patients in association with the deletion of the tumor suppressor genes *CDKN2A*/*B* [31], *RCBTB2*-*LPAR6* indicates a partial *RB1* loss as part of a larger deletion [32], and *DLEU2*-*SPRYD7* indicates the deletion of the miR-15a/16–1 cluster (Fig. 2). The deletions were confirmed by WGS SV and CNV calls in the respective patients. Since WTS is not limited to the detection of already known chimeric transcripts, we also identified in total 57 putative novel fusion transcripts (Additional file 6: Table S4). Although the potential therapeutic consequences and functions are yet to be determined, multiple genes associated with cancer or implied in non-hematologic malignancies were found as fusion partner genes in our dataset (e.g. *CHD4*, *HOXA7*, *FOXO3*).

### CNV calling based on WTS data identifies relevant ploidy groups

For the remaining 134 BCP-ALLs with no subtype defining rearrangements, CNV calling was performed based on the WTS data to identify relevant ploidy groups for further subclassification (Fig. 1). Here, the CNVkit algorithm was used to identify patients with high hyperdiploidy or low hypodiploidy/near-triploidy (Patients and Methods). The algorithm correctly identified 17 (94%) low hypodiploid/near-triploid ALLs and 12 (80%) high hyperdiploid ALLs as defined by WGS, arrayCGH, and FISH (Additional file 7: Table S5). One case was misclassified as high hyperdiploid, but is most likely a near-triploid ALL, according to the WGS data. The algorithm missed 1 hypodiploid/near-triploid ALL with low blast count (20%), 3 high hyperdiploid ALL, 1 near haploid ALL, and 1 iAMP21 ALL. However, the resolution of the algorithm might be too low to reliably detect iAMP21. We thus, analyzed the expression of *DYRK1A* and *CHAF1B* that have recently been associated with iAMP21-positive ALLs [33]. The expression of both genes was indeed heightened in the iAMP21 case (Additional file 2: Fig. S2A). Based on this classification, 103 (49%) BCP-ALL samples of our cohort have no established abnormalities and are further referred to as BCP-ALL ‘other’.

### *BCR*-*ABL1*-like signature identification by WTS

A compilation of the various published gene lists [34–36] was used to test for their ability to differentiate between *BCR*-*ABL1* positive and *BCR*-*ABL1* negative cases in our cohort. A final list of 26 genes with the highest variation between *BCR*-*ABL1* positive and *BCR*-*ABL1* negative cases and the reference profiles of 41 *BCR*-*ABL1* positive and 65 *BCR*-*ABL1* negative cases (Additional file 8: Table S6) were used to classify the 103 BCP-ALL ‘other’ cases into the *BCR-ABL1*-like and non *BCR-ABL1*-like groups, based on minimal distance. Twenty eight cases were classified as *BCR-ABL1*-like and the remaining 75, as non *BCR-ABL1*-like. Recently, a targeted RNA-Seq panel of 38 genes was published to identify adult BCP-ALL pts. with *BCR*-*ABL1*-like characteristics [37]. The application of this gene panel identified 30 *BCR-ABL1*-like cases, of which 28 (93%) were concordant with the group classification, by the list of 26 genes. Hence, the concordant subset of 28 samples was assigned to the *BCR-ABL1*-like subtype (Additional file 9: Table S7).

### Characteristics of *BCR-ABL1*-like and non *BCR-ABL1*-like cases

There were no significant differences in baseline characteristics such as age, gender, and ALL phenotype between *BCR-ABL1*-like and non *BCR-ABL1*-like patients (Additional file 3: Table S1), but for an elevation of white blood cell counts in *BCR-ABL1*-like patients (59.03 × 10^9^/L vs 25.18 × 10^9^/L, *P* = 0.025). Among the BCP-ALL ‘other’ cases, *CRLF2* showed a clear bimodal expression (Additional file 2: Fig. S2B), with a significant higher *CRLF2* expression in the *BCR-ABL1*-like group, as compared to the non *BCR-ABL1*-like cases (logFC 5.17, *P* < 0.0001). The high *CRLF2* expression could either be linked to the occurrence of a *CRLF2* rearrangement or *CRLF2* mutations. Only one case with a high *CRLF2* expression was assigned to the non *BCR-ABL1*-like group. In addition, a significant enrichment of *JAK2* mutations (mainly c.2047A > G) could be observed in the *BCR-ABL1*-like group (42% vs 0%, *P* < 0.001), whereas the non *BCR-ABL1*-like group carried a higher proportion of *NRAS/KRAS* (28% vs 4%, *P* = 0.007), *PAX5* (c.239C > G, 8% vs 0%, *P* = 0.12) and *TP53* (8% vs 0%, *P* = 0.12) mutations (Additional file 9: Table S7). Fusions involving *PAX5*, *CRLF2*, and tyrosine kinases were exclusively found in the *BCR-ABL1*-like group. All samples with detected *NUTM1*, *HLF*, and *ZNF384* fusion transcripts were assigned to the non *BCR-ABL1*-like group and, hence, could be further subclassified based on these genetic alterations. WGS data showed that 34% of the BCP-ALL ‘other’ cases harbored a deletion in *IKZF1,* and as expected, these deletions were significantly more common in the *BCR-ABL1*-like group (61% vs 24%, *P* < 0.001). A similar trend could be observed for *RB1* deletions (WGS data, 18% vs 4%, *P* = 0.019). In contrast, deletions of the tumor-suppressor gene *CDKN2A* (WGS data) were fairly common amongst both groups (32% vs 44%), and were not enriched in *BCR-ABL1*-like or non *BCR-ABL1*-like cases (Additional file 9: Table S7).

### A multi-modal approach is superior to a classification based on gene expression profiles alone

Most genetic alterations in ALLs are also associated with specific gene expression profiles, providing the basis for expression-based classification approaches such as ALLsorts (https://github.com/Oshlack/AllSorts). Hence, for BCP-ALL patients, we compared our results from the multi-modal approach to the ALLSorts classifier (see Patients & Methods; Additional file 10: Table S8). The ALLSorts classifier returns a matrix with per sample probabilities for each subtype. For the comparison, only the highest subtype probability was considered for each sample. The ALLSorts predictions were grouped into; unclassified (= BCP-ALL ‘other’; probability < 50%), low confidence (50–80% probability), medium confidence (80–90% probability), and high confidence (probability > 90%) calls.

**Fig. 3:**
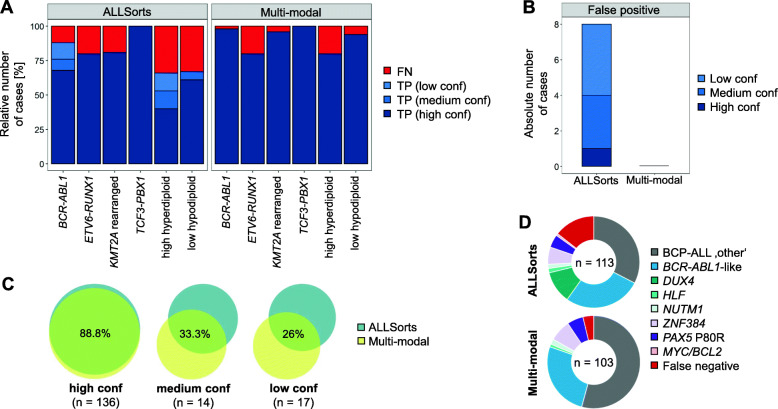
Performance comparison between the multi-modal approach and ALLSorts. Accuracy comparison for BCP-ALL patients harboring entity-defining fusion transcripts or abnormalities in chromosome number (**a**) and the number of false positive calls (**b**). **c** Overlap in label class for different confidence levels (low: 50–80% probability; medium: 80–90% probability; high: > 90% probability). **d** Breakdown of the identified BCP-ALL ‘other’ cases by both approaches. Conf: confidence; FN: false negative; TP: true positive

For the fusion transcript and ploidy based WHO subgroups, the ALLSorts classifier achieved an overall accuracy of 86%, compared to 97% of our stepwise approach. The ploidy groups had the highest number of false negative calls, and less than 50% of the high hyperdiploid cases were called with high confidence by ALLSorts (Fig. 3A). Although the single iAMP21 case could be identified by gene expression as mentioned above, it was not identified as such, by ALLSorts. The ALLSorts classifier also made 8 false positive calls with different confidence levels, compared to zero false positive calls of the fusion calling (Fig. 3B). It was also evident that the overlap of assigned class labels between ALLSorts and the multi-method approach dropped from 89 to 26% with decreasing probability values (Fig. 3C). Due to the higher number of false negative calls, ALLSorts assigned more cases to the BCP-ALL ‘other’ group compared to the multi-modal approach (113 vs 103 Fig. 3D). The approaches agreed on 26 of the *BCR-ABL1*-like cases, while ALLSorts misclassified 3 *BCR*-*ABL1* cases as *BCR-ABL1*-like. ALLSorts classified 15 patient profiles as *DUX4* rearranged (Fig. 3D). However, neither *DUX4* fusion transcripts nor *DUX4* expression (WTS data) or the IGH-*DUX4* structural variants (WGS data), could be identified in those cases. Nevertheless, compared to BCP-ALL ‘other’ cases not classified as *DUX4* rearranged, an overexpression of *DUX4* target genes such as *PCDH17* (logFC 7.51, *P*-value < 0.0001), *PDGFRA* (logFC 5.65, P-value < 0.0001), and *AGAP1* (logFC 5.52, P-value < 0.0001) could be observed. ALLSorts correctly identified 6 samples with a *PAX5* c.239C > G mutation. However, one case of *PAX5* c.239C > G was missed, and in one case the additional high hyperdiploidy was not detected. Both cases were correctly identified by the stepwise approach. ALLSorts correctly identified all cases harboring a *ZNF384* or *NUTM1* fusion transcript and one case with a *HLF* fusion transcript as detected by the multi-modal approach. One case was labeled as a MYC/BCL2 double-hit BCP-ALL by ALLSorts, but solely carried a *MYC* translocation (WGS data).

## Discussion

Genetic aberrations in ALL are structurally diverse, and currently detected by a variety of diagnostic assays. The aim of this study was to compile a diagnostic workflow to establish whole transcriptome RNA sequencing as a reliable, comprehensive, and efficient assay for ALL diagnostics. We demonstrated that typical genetic alterations can be identified with high accuracy, while at the same time the unbiased assessment of the transcriptome also allows the identification of potentially new targets in patients, where these genetic aberrations are absent. Our results further suggest that careful selection of the algorithms for each molecular type is beneficial for accurate sample classification.

We demonstrated that samples could efficiently be classified in a stepwise approach (Fig. 1). As previously shown [38], BCP-ALLs were characterized by a homogenous *CD19* gene expression, whereas T-ALLs could be identified by *CD3D* and *CCR9* expression. Multiple entity-defining fusion transcripts are known in BCP-ALLs (*BCR-ABL1*, *KMT2A-AFF1*, *TCF3-PBX1*, *ETV6-RUNX1*), and a reliable detection is mandatory for every diagnostic workflow. The applied fusion calling procedure identified 97% of the fusions, as detected by gold standard techniques, which is in line with previous RNA-Seq studies in pediatric ALL cohorts that reported detection rates between 91 and 97% [3, 39–41]. The number of true positive calls can be increased by considering the overlap of different callers [42], while simultaneously reducing the number of false negative ones. In this study, we used three different algorithms, with only 68% of the risk-stratifying fusion events being called by all the three algorithms (Additional file 6: Table S4), advocating the combined approach. Two fusion transcripts were missed most likely due to the low fusion transcripts expression, which are very difficult to detect [3, 40] and cannot be rescued by this approach. Here, only a greater sequencing depth could solve this issue.

Beside subtype defining rearrangements, other previously described translocations could be identified, including 8 rearrangements involving *ZNF384*, which were recently described to constitute a new molecular subtype of BCP-ALL ‘other’ with a good response to prednisone and conventional chemotherapy [43–45]. Our study showed that WTS is especially beneficial to identify cytogenetically cryptic events (e.g. *EP300*-*ZNF384*, *PAX5* fusion transcripts, *SET*-*NUP214*, etc) and unknown or divers fusion partners, in an unbiased and cost-effective way. In addition, we identified multiple recurrent read-through events indicative of gene deletions, frequent in ALL (e.g. *CDKN2A*/*B*, *RB1*, *MIR15A*/*16–1*), which were exclusively called by STAR-Fusion and arriba. To the best of our knowledge, two of these events have not been described before, whereas the *MTAP*-*ANRIL* fusion has been identified in a melanoma cohort, in the context of *CDKN2A*/*B* deletions [31]. *CDKN2A*/*B* deletions have been associated with poor prognosis and it has been suggested to declare them as an additional B-ALL subgroup [46]. Moreover, WTS identified 57 putative novel fusions with the majority occurring only in a single patient; similar to the findings in a study of pediatric ALL [39]. As all these fusions were detected only once, the putative role in ALL pathogenesis and their diagnostic and prognostic potential has to be determined by combining data from several studies. However, fusion transcripts involving genes with a large number of pseudogenes (e.g. *DUX4*) or highly variable genomic regions (e.g. IGH gene locus) are still challenging to detect with most fusion calling algorithms but this might be improved by their continuous optimization.

Estimating abnormalities involving the chromosome number, plays a major role in ALL classification and prognostication. While ALL with high hyperdiploidy is associated with a favorable prognosis, ALL with low hypodiploidy shows a poor outcome [15, 47]. Due to the interplay of multiple regulating factors, inferring copy number changes from WTS data is rather challenging [48]. In our study, the determination of ploidy groups had the highest error rate, missing 5 cases, compared to the ones from WGS, arrayCGH, and FISH. However, CBA missed 4 low hypodiploid/near-triploid cases due to low in vitro proliferation, which were identified based on WTS data and confirmed by WGS. The resolution of the applied algorithm was too low to identify the iAMP21 case, or to reliably detect single gene deletions. While in the case of iAMP21 the gene expression could be used for the classification, the same did not hold true for gene deletions, as mentioned in a previous study [3]. Here, the analysis of isoforms and differential transcript usage might provide the needed insights, but these analyses were out of the scope of this work. In addition, a larger set of iAMP21-positive cases is needed to proof the validity of *CHAF1B* and *DYRK1A* gene expression as biomarkers for the presence of iAMP21, since our cohort included only one such case.

*BCR*-*ABL1*-like ALLs are one of the most relevant new subgroups due to the potential benefit for treatment with tyrosine kinase inhibitors, as further underlined by the poor outcome for this ALL subtype on conventional treatment strategies [49]. False negative results are rare, and the actual risk and clinical impact in such cases is unknown [50]. Various gene lists have been published in the literature [34–36] for gene expression profiling, with only partial overlapping between the lists and the resulting classification. For WTS we identified a list of 26 genes to identify *BCR*-*ABL1*-like cases. The majority of the genes (65%) were also present in the recently published list of a targeted RNA-Seq panel [18], resulting in an overlap of 93% in classification results. We only characterized 13% of our BCP-ALL cohort as *BCR*-*ABL1*-like cases, which is below the typical range of 24–33% [37, 51, 52]. However, 67% of the patients from our BCP ALL cohort fall into the adult or elderly age group, and it has been shown that the frequency of *BCR*-*ABL1*-like cases declines with age, with an incidence just of between 7 and 20% for adults and elderly patients [53, 54].

In line with published data, cases with *CRLF2* rearrangements and *IKZF1* deletions were significantly more common in *BCR*-*ABL1*-like cases [49, 55]. One case with an *IGH*-*CRLF2* rearrangement and high *CRLF2* expression was not classified as *BCR-ABL1*-like ALL. In pediatric ALL it has been reported that 5 to 10% of patients with *CRLF2*-rearranged ALL have distinctly different gene-expression profiles without the kinase-activated signature [49]. In the *BCR-ABL1*-like subgroup we also identified 3 patients harboring a *P2RY8*-*CRLF2* fusion, which is associated with poor prognosis in children [56], and 3 patients with fusions involving *PAX5*. Cases harboring *PAX5* or *CRLF2* fusions have been proposed as an independent subgroup in BCP-ALL [2]. 42% of *BCR*-*ABL1*-like cases carried a *JAK2* mutation, which is comparable to previous studies that reported 27–57% of mutated *JAK2* [37, 51, 52]. Among the non *BCR-ABL1*-like subgroup, we identified various cases with *PAX5* (c.239C > G) mutations, along with cases harboring *ZNF384*, *HLF* or *NUTM* rearrangements, all of which have been recently identified as new BCP ALL subgroups [2, 45]. The ALLSorts algorithm also identified a *DUX4* transcriptional signature in 15 cases, but no indication for *DUX4* fusion transcripts could be identified in them, as these fusions were predominantly described in pediatric and AYA ALL (adolescent and young adults) [57], and our cohort included only a small number of young patients. However, it is well known that fusion transcripts involving *DUX4* are difficult to detect with standard fusion calling pipelines and gene expression profiling might be superior in these instances.

Further comparison between the gene expression profile based ALLSorts classifier and our stepwise approach, showed a good concordance for high confidence calls (Fig. 3C). However, our approach applies optimized algorithms for the different molecular types, resulting in an overall more precise classification, with superior performance for the identification of ploidy groups and a reduced number of false positive calls.

## Conclusion

In summary, our study demonstrates that WTS can be used to reliably classify ALL patients with a single assay and is superior to conventional methods in cases which lack entity-defining genetic abnormalities. With the decrease in sequencing costs, the integration of WTS in routine diagnostics of ALL patients seems feasible, however, requiring the definition of standardized quality parameters and data analysis workflows to enable reproducibility and comparability between laboratories.

## Supplementary Information

**Additional file 1: Figure S1.** Gene expression of the B-cell and T-cell lineage**.** t-SNE plot of gene expression of selected marker genes (a-c, perplexity: 25). d) Correlation between *CD10*/*CD1A* gene expression and CD10+/CD1A+ cells as determined by immunophenotyping. Colors correspond to lineage, immunophenotypic subtype or expression as indicated by the plot legends.

**Additional file 2: Figure S2.** Expression of selected genes. a) *CHAF1B* and *DYRK1A* expression of BCP-ALL patients (*n* = 104) without risk-stratifying fusions or abnormal chromosome number. b) *CRLF2* expression of BCP-ALL ‘other’ patients (*n* = 103).

**Additional file 3: Table S1.** Patient data.

**Additional file 4: Table S2.** Allele concordance scores between WTS and WGS SNP profiles.

**Additional file 5: Table S3.** Gene expression of known lineage markers.

**Additional file 6: Table S4.** List of called fusion transcripts in both lineages.

**Additional file 7: Table S5.** Copy number variations called by CNVkit for WTS data.

**Additional file 8: Table S6.** Expression of BCR-ABL1-like signature genes.

**Additional file 9: Table S7.** Characteristics of BCR-ABL1-like and non BCR-ABL1-like cases.

**Additional file 10: Table S8.** Comparison to ALLSorts algorithm.

## Data Availability

All data analysed during this study are included in this published article and its supplementary information files. Additional datasets are available from the corresponding author on reasonable request.

## Abbreviations

ALL: Acute lymphoblastic leukemia
AYA: Adolescent and young adults
CBA: Chromosomal banding analysis
CNV: Copy number variation
FISH: Fluorescence in situ hybridization
iAMP21: Intrachromosomal amplification of chromosome 21
SNP: Single nucleotide polymorphism
SV: Structural variant
WGS: Whole genome sequencing
WHO: World Health Organization
WTS: Whole transcriptome sequencing
