# Supplementary figures and images for: Clinical application of whole transcriptome sequencing for the classification of patients with acute lymphoblastic leukemia
Source: BMC Cancer. 2021 Aug 2;21:886. doi: 10.1186/s12885-021-08635-5 (PMC8330044; doi:10.1186/s12885-021-08635-5)

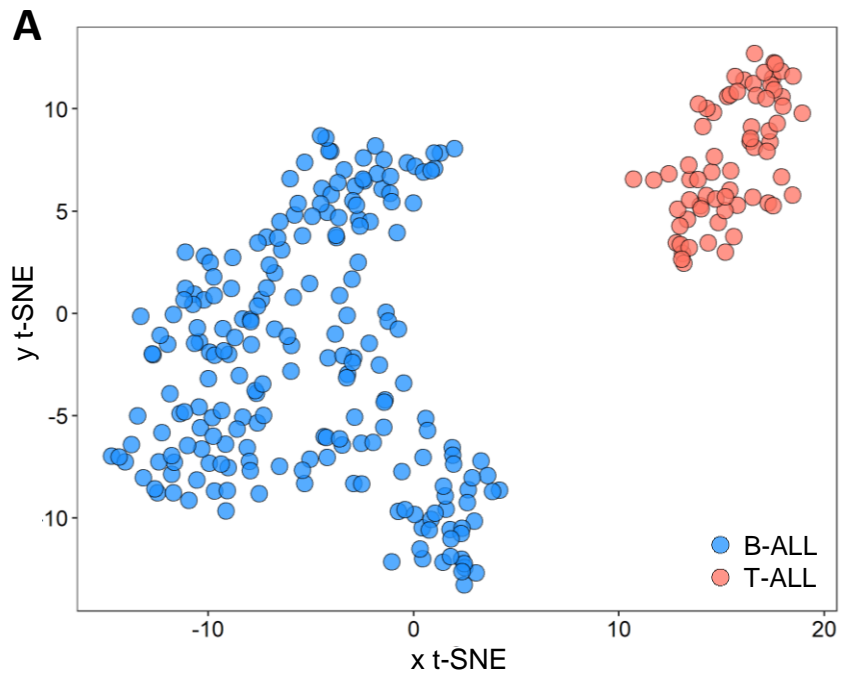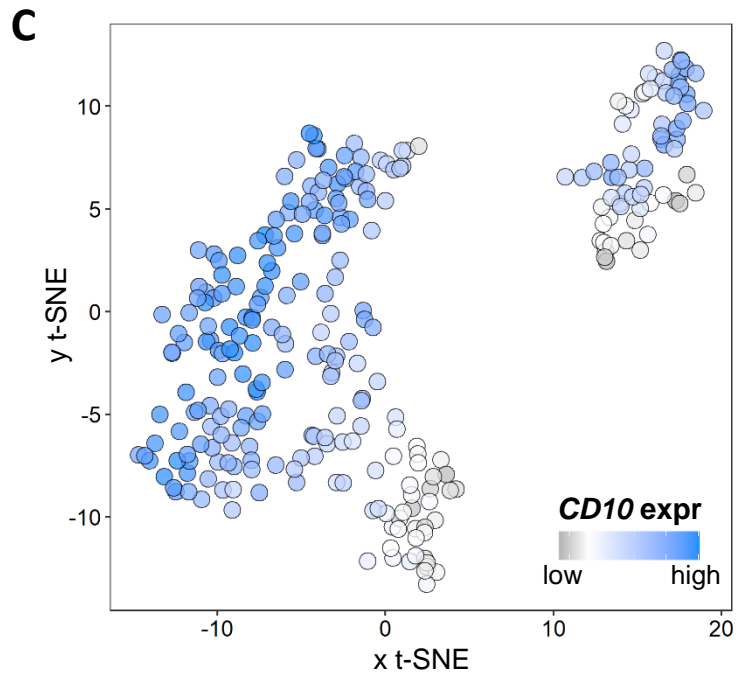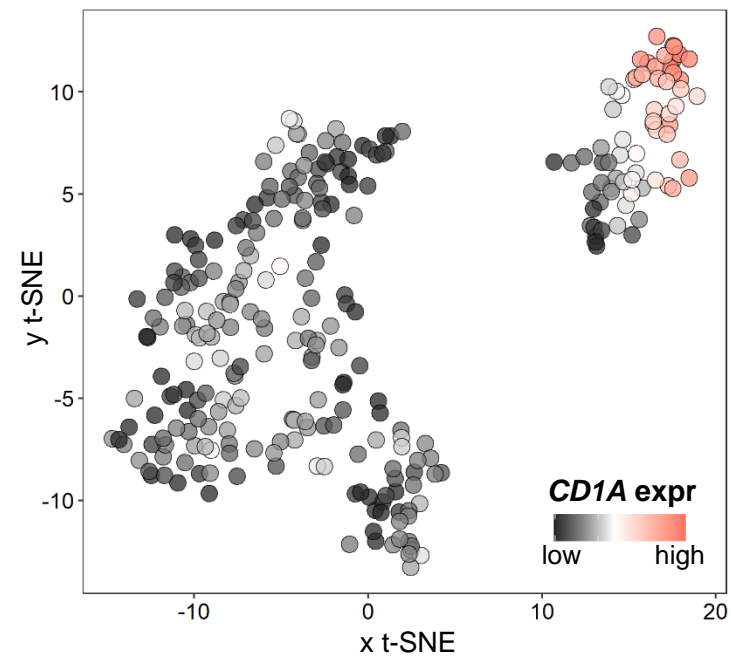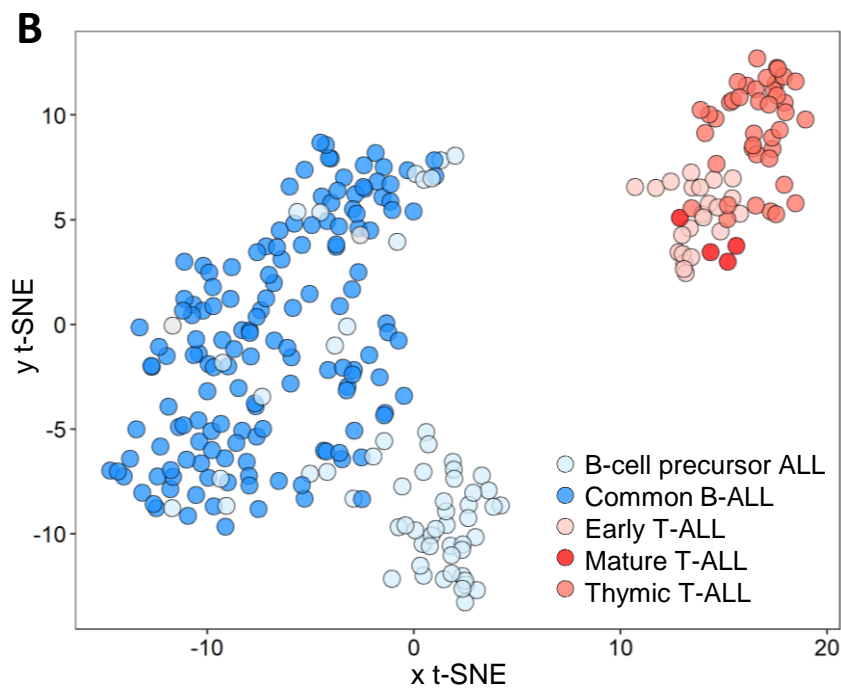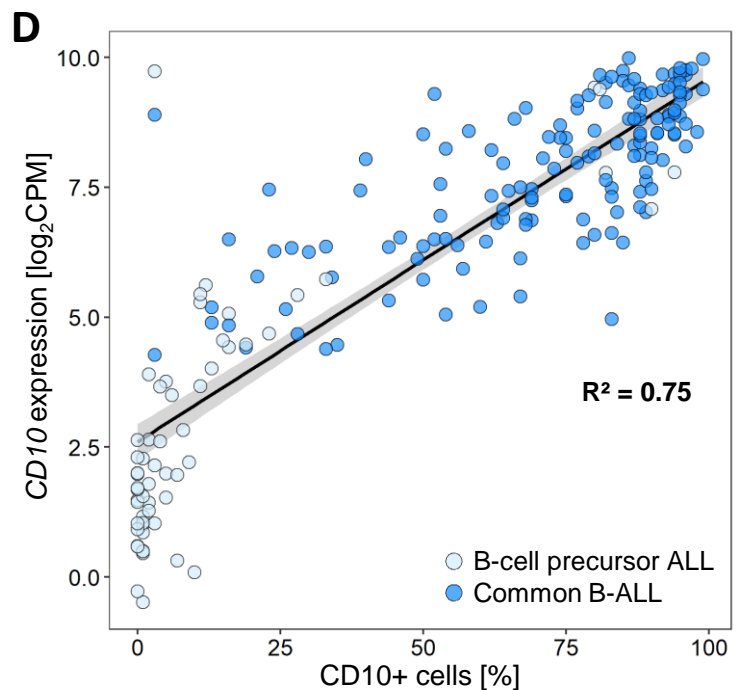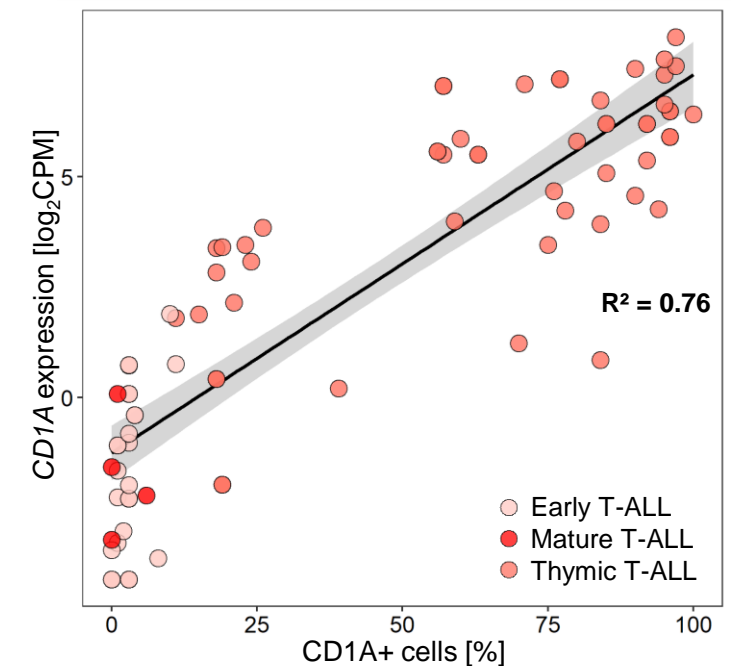

Supplement: Supplementary file 1 — Additional file 1: Figure S1. Gene expression of the B-cell and T-cell lineage. t-SNE plot of gene expression of selected marker genes (a-c, perplexity: 25). d) Correlation between CD10/CD1A gene expression and CD10+/CD1A+ cells as determined by immunophenotyping. Colors correspond to lineage, immunophenotypic subtype or expression as indicated by the plot legends. [file 12885_2021_8635_MOESM1_ESM.pdf]

**A**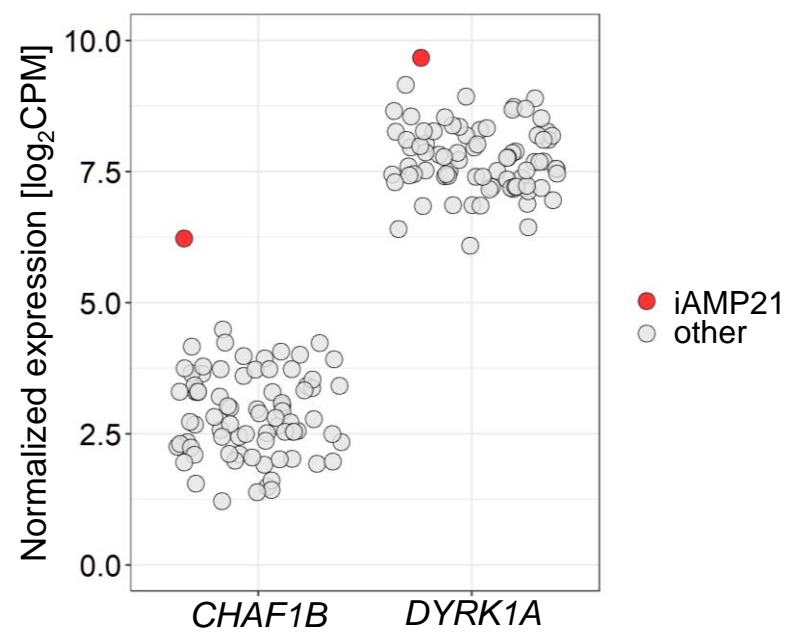**B**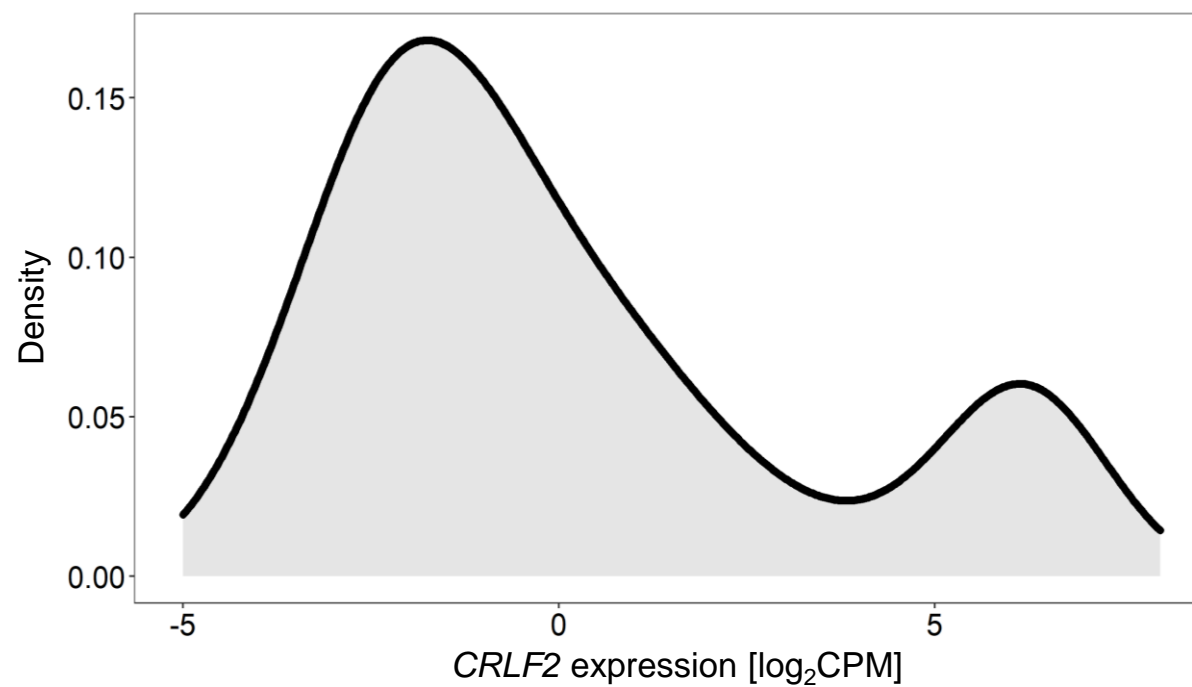

Supplement: Supplementary file 2 — Additional file 2: Figure S2. Expression of selected genes. a) CHAF1B and DYRK1A expression of BCP-ALL patients (n = 104) without risk-stratifying fusions or abnormal chromosome number. b) CRLF2 expression of BCP-ALL ‘other’ patients (n = 103). [file 12885_2021_8635_MOESM2_ESM.pdf]
